# Voice Recovery in a Patient with Inhaled Laryngeal Burns

**Published:** 2019-01

**Authors:** Geun-Hyo Kim, Soo-Geun Wang, Yeon-Woo Lee, Soon-Bok Kwon

**Affiliations:** 1 *Department of Otorhinolaryngology-Head and Neck Surgery* * and Biomedical Research Institute* *, Pusan National University Hospital, Busan, * *South Korea* *.*; 2 *Department of Humanities, Language and Information, Pusan National University, Pusan, South Korea.*

**Keywords:** Dysphonia, Inhalation Burns, Larynx, Laryngoscopy

## Abstract

**Introduction:**

Laryngeal burns cause long-term voice disorders due to mucosal changes of the vocal folds. Inhalation injuries affect voice production and result in changes in the mucosal thickness and voice quality.

**Case Report:**

A 47-year-old woman was transferred to our department with laryngeal burns sustained during a house fire. On laryngoscopic examination, mucosal waves of both vocal folds were not visualized due to the injury caused by inhalation of high-temperature toxic smoke. Hence, voice analysis, laryngoscopic examinations, and high-speed videoendoscopy (HSV) were performed to evaluate vocal fold vibrations. An absence of mucosal waves and a breathy and strained voice with a severe grade were noted. We report that voice quality was recovered to close to the normal state through multiple treatments such as medication, voice therapy, and counseling.

**Conclusion:**

This paper presents the unique case of a patient with laryngeal burns, in which vibrations of the vocal folds were observed using laryngoscopic examination and HSV. Voice samples before and after treatment were also analyzed. By observing the vibration pattern of the injured vocal fold, it is expected that appropriate diagnosis and treatment planning can be established in clinical practice.

## Introduction

Inhalation burns occur in 30% of all patients with burns, while 20% of patients with inhalation injury have extensive laryngeal injury. A further 7% of patients with inhalation injury have both laryngeal and tracheobronchial injuries (1). High-temperature smoke inhalation causes injury to the upper and lower airways. Through the airways and the larynx, pathological changes in the internal area are noticeable (2). Dysphonia is present in as many as 70% of patients with inhalation injuries, 16 to 25 years after the initial injury (3-5). The soft tissue of the vocal folds affects the voice quality due to changes in the elasticity and edema of the larynx. Thus, movements of the vocal folds and mucosa membrane waves of the epithelial layer are reduced, making it difficult to produce voiced sounds. The initial treatment of patients with various types of burn remains an important issue in burn intervention and is an ongoing controversial discussion (6). Initial treatment of laryngeal burn patients is focused on life support, such as morphologic laryngeal changes and tracheotomy (3). Treatments for dysphonia caused by laryngeal burns are initiated after first aid treatment (7). If evaluation and treatment of dysphonia after a laryngeal burn are performed immediately, the medical team can establish an optimal treatment plan.The current study is a unique report that attempts to evaluate vocal fold vibrations in a patient with laryngeal burns following an inhalation burn injury through multiple analyses, including laryngoscopic examinations, high-speed videoendoscopy (HSV), and acoustic analysis. Related literature has also been reviewed. This study was approved by the institutional review board of Pusan National University Hospital.

## Case Report

**Fig 1:**
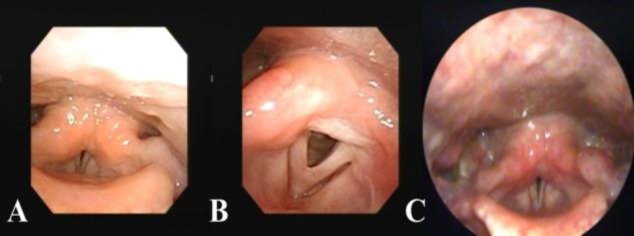
Images of laryngoscopic examinations. A: Laryngoscopy at the first visit; B: Laryngoscopy at 6 months after the inhalation burn; C: Laryngeal videostroboscopy at the first visit

A 47-year-old woman was brought to our department with laryngeal burns sustained during a house fire. She wished to confirm the changes in the larynx. She was also counseled to relieve post-traumatic stress. During the first visit, the patient presented with a severe breathy voice, and was noted to have a cough. Although laryngeal edema was present, the airway was secure. Laryngeal movement was restricted during phonation. Laryngoscopic examinations showed movements of the vocal folds during respiration, but could not evaluate the vibratory patterns of the vocal folds during phonation because of the non-vibrating portion (Fig.1). 

**Fig2:**
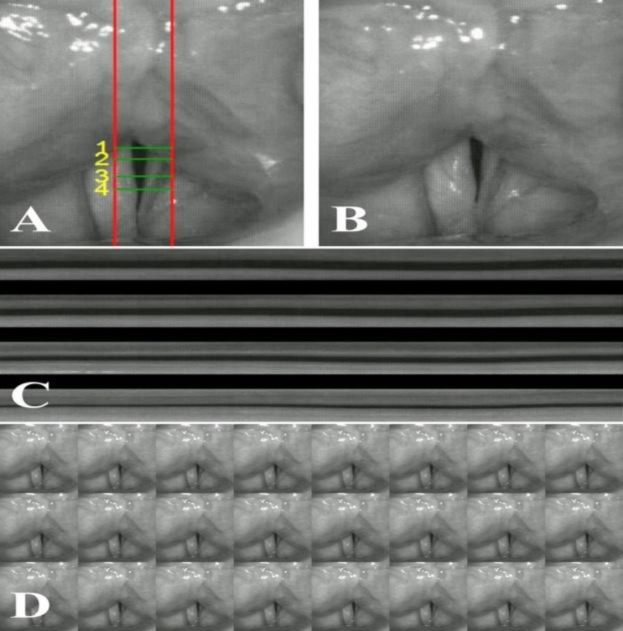
Multiple analyses of vocal fold vibrations in a patient with laryngeal burns. A: HSV display; B: 2D DKG; C: DKG; D: Sequential HSV images

Also, non-periodic vocal fold vibrations were invisible on laryngeal examination. HSV was used to observe the micro-vibrations in the non-vibrating portion. This means that the mucosa of the entire vocal folds was changed to a condition of increased stiffness. The absence of mucosal waves was also confirmed by digital kymography (DKG) and two-dimensional kymography (2D DKG) (8-10) (Fig.2). 

Acoustic analysis was performed to quantify the voice quality. Cepstral analysis of the voice signal was performed by analysis of dysphonia in speech and voice (ADSV; model 5109 v.3.4.2; KayPENTAX, Montvale, NJ). The results of cepstral peak prominence (CPP), low-to-high frequency spectral ratio (L/H spectral ratio), and mean CPP F0 are presented in Table 1. The CPP is a measure of the degree of harmony within voice signals. The L/H spectral ratio represents the mean ratio of the signal energy below 4,000 Hz to the energy above 4,000 Hz for the selected voiced data frames (11). Additionally, an auditory perceptual assessment using the GRBAS scale was also performed. The GRBAS scale, which assesses the overall degree of dysphonia, as well as rough, breathy, asthenic, and strained parameters, is widely used in voice research and clinical practice (12,13). Initially, medication and voice therapy were planned as the patient complained of throat pain and dysphonia. An expectorant, bronchodilator, and antibiotic were prescribed for foreign body sensation and pain in the neck. Subsequently, intensive voice therapy was commenced for voice production, with effective glottal closure. Voice therapy techniques, such as vocal function exercises, accent method, and inhalation phonation were used ***(***14***). ***

Additionally, an intracordal injection was considered as a second intervention for glottal contact. It was expected that the quality of the vocal fold mucosa and voice would improve based on the treatment plan.

Fortunately, there was no need to perform the injection because the voice quality was noted to have improved after 6 months of follow-up, and it was decided to administer medications alone and observe the patient without any additional treatment (Table.1).

 An anterior web was noted to form during the recovery process, but this did not affect the patient’s breathing.

**Table1:** Results of the acoustic and auditory perceptual assessment

	**First visit**	**After 6 months**
CPP	0.35	9.85
L/H spectral ratio	9.52	23.56
Mean CPP F0	232.78	208.82
GRBAS	G3R1B3A0S3	G1R1B1A1S0

## Discussion

The patient with laryngeal burn injuries showed voice recovery through multiple treatments. Although the absence of vocal fold vibration and severe voice quality were noted at the first visit, we confirmed phonatory recovery after 6 months. The clinical highlights were as follow. First, laryngoscopic examinations cannot visualize the vocal fold vibration in patients with laryngeal burns, whereas HSV can visualize micro-vibrations or the non-vibrating portion of the damaged vocal fold. As a result, decreased mucosal waves were confirmed by HSV. This supports the existing hypothesis that inhalation burns will seriously damage the mucous membranes and cause changes in the voice. Second, we reported that a multi-treatment approach, including medication, voice therapy, and counseling, could be effective in this case.

Previous studies have used laryngoscopic examinations to confirm the extent of the injury to the upper airway, not for observation of the vocal fold vibrations. It was difficult to accurately evaluate fine vibrations of the vocal folds and aperiodic voices. Based on the patients’ report and laryngoscopic findings, patients were classified into three categories (mild, moderate, and severe) (7). Clayton, Kennedy, and Maitz suggested that performing videoendoscopy in all tracheostomized burn patients would be beneficial for a more accurate assessment of laryngotracheal pathology (15).

Focusing on efforts to overcome emergency situations, the laryngoscopy was used to observe and to aid in securing the airway. By giving priority to securing the airway as the initial treatment in patients with inhalation burns, laryngeal edema and lung injury were confirmed. Casper et al. reported that decreased mucosal waves, contralateral edema, stiffness, phase abnormality, and glottal chink were revealed through videostroboscopy, which provides useful information in determining the cause of dysphonia (3). Valdez et al. reported the use of laryngoscopy to identify abnormal findings in the posterior glottal area, and further acquired information on voice disorders using laryngeal videostroboscopy after extubation (4). There was no mention of any changes in the non-vibrating portion. Laryngeal examination systems that can observe vocal fold vibrations provide a unique opportunity to associate anatomical abnormalities with vocal function.

**Fig 3:**
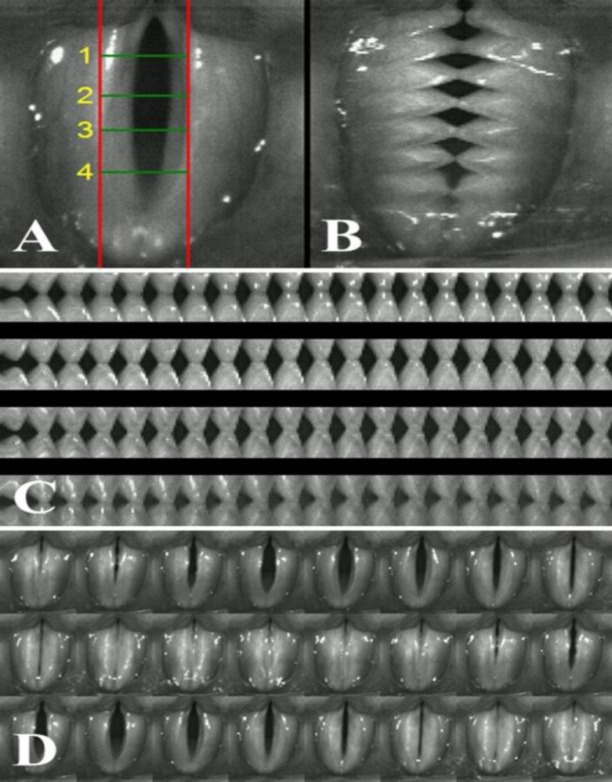
Multiple analyses of vocal fold vibrations in a normal subject (author; male, 33 years of age).

In this study, we focused on the voice change experienced by the patient after the acute phase of inhalation injury. We confirmed that the changes in mucosa occurred due to smoke and heat stimulation. The entire vocal fold showed the non-vibrating portion of the vocal fold. On DKG analysis, the anterior–posterior portion of the vocal fold was divided into quadrants, and it was confirmed that no kymograms were observed in all four scan-line positions. On 2D DKG analysis, no diamond-shaped glottal opening of the entire vocal fold was observed. This result was also confirmed by sequential HSV images. Additionally, breathy and strained voices were produced by compensatory action of incomplete closure and mucosa membrane change. Cepstrum analysis was performed to quantify the voice quality. Values of CPP, L/H spectral ratio, and mean CPP F0 were changed positively after multiple treatments (Table.1). HSV assessment after voice therapy was not performed because the patient refused further evaluation due to discomfort of oral videoendoscopy. Although we did not present HSV after voice therapy, we generated the image of a normal vocal fold vibration pattern to help in the understanding of the 2D DKG (Fig.3). 

Based on the results of the cepstral analysis, we predicted that the vibratory pattern of the patient’s vocal folds was restored to the normal vibratory pattern after multidisciplinary treatment. At 6 months post-injury, the patient’s voice had improved (from G3R1B3A0S3 to G1R1B1A1S0). The results of Hogg et al. were similar to our results. Voice therapy and medication were performed, and as a result, recovery of vocal disorders was reported (2).

Laryngeal burns result in impaired laryngeal structure and voice disorders. Therefore, various specialists, including specialists in burns treatment, laryngologists, and speech language pathologists, should cooperate in the management of burns and the subsequent rehabilitation (16,17). Inhalation burns are life-threatening and usually need long-term recovery and rehabilitation. In terms of voice rehabilitation, it is necessary to evaluate vocal fold vibrations more precisely in order to achieve an effective voice output. The degree of mucosal change on the surface of the vocal folds should be checked to determine the prognosis and treatment approach.

## Conclusion

In the case of a patient with laryngeal burns following an inhalation burn injury, the patient’s voice was recovered by appropriate diagnosis and a treatment plan. It is important to observe vocal fold vibrations using multiple assessments because an abnormal voice can be produced under various conditions of the vocal fold mucosa. Since the degree of the inhalation injury differs in each case, this approach makes it possible to determine more accurately the state of the vocal fold and to provide an appropriate treatment plan in patients who complain of voice changes after a laryngeal burns injury.
